# Milk Analysis and Infant Feeding

**Published:** 1885-12

**Authors:** 


**Milk Analysis and Infant Feeding.** By ARTHUR V. MEIGS, M.D. Published by P. Blakiston & Co. 95 pp.; 12mo. Price, $1.00.

This is an excellent little work upon a subject of the greatest importance. As civilization, so-called, advances, it becomes more and more necessary to resort to the artificial feeding of infants. This is especially the case in cities where the difficulties of obtaining suitable milk for children are often very great. Many thousands of infants die every year because those who care for them do not know what to feed them. The author devotes the first half of his work to the description of methods of analysis, after which he discusses artificial feeding, recommending milk sugar as an addition to cow's milk. This procedure has been severely criticised, and we think with good reasons, by Dr. A. Jacobi, in his article published in *Buck's Hygiene:* "Milk sugar rapidly turns to lactic acid, and an excess accumulates in the stomach. Milk sugar causes coagulation of protein, rendering it indigestible; it loosens alkalies and lime from its phosphoric combination, thereby eliminating phosphoric acid before the proper time, and giving rise to diarrhœa and rhachitis."

The work shows careful research, and is a real addition to the literature of this subject. We take pleasure in recommending it to our readers.

